# Dropping Out of School: Explaining How Concerns for the Family’s Social-Image and Self-Image Predict Anger

**DOI:** 10.3389/fpsyg.2020.01868

**Published:** 2020-08-04

**Authors:** Nicolay Gausel, David Bourguignon

**Affiliations:** ^1^ Faculty of Health and Welfare, Østfold University College, Fredrikstad, Norway; ^2^ PErSEUs, Sciences Humaines et Sociales, Université de Lorraine (Metz), Nancy, France

**Keywords:** self, social, image, dropping out, school, anger, stigma, family

## Abstract

As dropping out of school is considered a violation of moral norms, the family associated with the drop out can react with anger directed toward the pupil or with anger directed at others that might know of the drop out. In our vignette study (*N* = 129), we found that anger at others and anger at the pupil were significantly higher if our community participants imagined a drop out from a vocational education rather than a general education. As expected, anger directed at others was fully explained by a concern for the family’s social-image (i.e., a concern for condemnation by others), while anger directed at the former pupil was fully explained by a concern for the family’s self-image (i.e., a concern for their moral self-image). Thoughts for how to better understand family reactions in relation to drop out are discussed.

## Introduction

«Hvorfor er det sånn at jeg må være flau, hvorfor må de som har valgt et yrkesfaglig program bli stemplet som dumme, teorisvake og skoleleie?» [“Why do I have to be embarrassed, how come those who choose vocational education are labeled as stupid, theoretically weak and sick of school?”]

- Girl, 17 years, vocational student (interviewed in Aftenposten, 2015, translated by us).

Even though most educational programs are organized in order to support integration into the social and professional world (Beblavy et al., 2011), some pupils decide to drop out of these educational programs. As a drop out has the potential to be perceived by others as a violation of the expected egalitarian integration path (Van Hoorn and Maseland, 2013), their norm violating decision is often met with stigmatizing condemnation by the larger community (Weiner et al., 1988; Dorn, 1993; Hebl et al., 2007; Gausel, 2014). As a response to condemnation, people sometimes respond with blame (Gausel, 2014) and anger (Gausel et al., 2018). However, little if nothing is known about the families’ reactions and especially whether families respond with anger if their son or daughter drops out of school.

In order to investigate whether families would respond with anger to condemnation for dropping out of school, we asked community participants to imagine how a family would react if their son or daughter dropped out of an educational program. We expected that the more our participants expressed that the family would be concerned about the moral self-image of the family, the more the family would direct anger at their son or daughter (i.e., the former pupil). In contrast, the more our participants expressed that the family would be concerned about the social-image of the family, the more the family would direct anger toward others that might learn of the drop out.

### Vocational Education: Dropping Out

In most western countries, the high-school (or upper secondary school) educational system consists of general education and vocational education. Even though most pupils choose vocational education in order to acquire a professional job qualification, the real-world citation by the 17-year old girl in the introduction demonstrates that vocational education has become to be viewed as a second-chance education (Karmel and Woods, 2008) for pupils falsely believed to be less intelligent and thus having “a lower level of general aptitude” (Arum and Shavit, 1995, p.188). Due to this stigmatized belief, vocational pupils are therefore seen to be suited for professional work, instead of the more “university-oriented” general education (Grootaers et al., 1999; Gausel, 2014). As a consequence of this stigma, many pupils within the vocational education report that they feel that others look down at them for following a vocational program (Spruyt et al., 2015).

As the educational system represents the egalitarian view that everyone deserves a fair chance of bettering their position regardless of their background (Beblavy et al., 2011), dropping out of the educational system represents “a serious problem, not only for the individual, the school system, and the community, but also for society.” (Christle et al., 2007, p. 325). Even though dropping out – in general – is understood as a problematic norm violation (Dorn, 1993), dropping out from a vocational education seems to be *more* problematic for the pupil and its family for at least two reasons: firstly, dropping out violates the social ascension belief that members of low status groups should climb the social ladder *via* the educational system (Festinger, 1954; Hauser et al., 2000). Secondly, as western people typically believe that one is responsible for one’s own fate (Bénabou and Tirole, 2004), a discontinuation of schooling violates the meritocracy belief that individuals should demonstrate perseverance (Lerner, 1980). Dropping out of a vocational education can therefore be perceived by the larger society as the pupil is entering a competitive labor market without formal means to partake (Christle et al., 2007). Thus, the pupil is risking unemployment and dependence on welfare benefits (Christle et al., 2007; King et al., 2010). As people generally react harshly toward norm violators (Crocker et al., 1998; Major and O’Brien, 2005; Täuber et al., 2018), dropping out of vocational education has the potential to cause considerable psychological distress, not only for the pupil (Dorn, 1993), but also for the family associated with the drop out (Gausel, 2014) as families are commonly seen as a group (Scabini and Manzi, 2011).

### Anger: The Role of the Self-Image and the Social-Image

According to Gausel and Leach (2011), a norm violation of this kind can be appraised in at least two main ways: firstly, as an indication that there is something morally defective with the family, since they allowed a violation of a societal norm (i.e., a threat to the moral self-image of the family) by failing to prevent the drop out, and thus, failing take advantage of the social ascension possibility and failing to demonstrate perseverance. Failures that are appraised as representing a threat to the self-image are often associated with anger directed at the self (Miller and Tangney, 1994; Gausel and Leach, 2011) or one’s in-group (Gausel and Leach, 2011). As it is well known that families represent a group and its members are group members (for discussions, see Scabini and Manzi, 2011), it is interesting to observe that on a family-related level, Gausel et al. (2016) found that participants appraising themselves as suffering from a morally defective self-image directed anger toward themselves as a consequence for their abusive behavior toward a family member. And Berndsen and McGarty (2012) found that majority group members reminded about moral failures committed by their group expressed anger at their own group in response to these failures. Similar to this, Gausel et al. (2012) found that the more their participants appraised their in-group moral failures as a threat to their in-group self-image, the more anger they directed toward their own group. Hence, in response to the current study, we expected that a concern for the self-image of the family as caused by the drop out would be predictive of self-directed anger.

Secondly, as there is a real risk that failures can draw condemning attention from others (Gausel and Leach, 2011), a drop out may pose a serious threat to the family’s social-image as respectable in the eyes of others. If such a threat to the social-image is appraised, people often react with anger directed at the others that can possibly come to condemn them for their failure (Gausel, 2013). In empirical support of this, a recent study on family therapy and reciprocal partner-violence, Zahl-Olsen et al. (2019) found that outburst of anger and violence toward the other was associated with appraised condemnation manifested through rejecting behavior from the other as well as criticism for failure. Gausel et al. (2018) found that the more victims of immorality feared that they would be condemned for their own perpetrating failures in a reciprocal conflict, the more they reacted with hostile anger toward others. In response to the current study, we expected that a concern for the family’s social-image would be predictive of other-directed anger.

In sum, there is ground to assume that being associated with dropping out of school can be appraised by a family as a threat to their self-image as dropping out symbolize the failure to demonstrate perseverance, as well as the failure to conform with the social ascension belief. This might very well predict anger directed at the responsible one, i.e., the pupil. That said, there is also ground to believe that the eyes of others are now critically resting on the family. Thus, being associated with dropping out of school represents a vivid threat to the social-image of the family, especially if they fear that these others get to find out about the failure. If so, the family might very well direct anger against these others.

### The Current Study

In order to test the above assumptions, we returned to a large-scale study where parts have previously been reported in a manuscript by Gausel (2014). However, none of the measures, and analyses and none of the correlations reported here in this manuscript have been examined or reported elsewhere. For the sake of clarity, we illustrate how the measures are used across the two manuscripts in Table 1.

**Table 1:** Illustration of the measures used.

Variables	MS 1 (published in SPE)	MS 2 (current)
Embarrassing failure	x	
Felt rejection	x	
Blaming the school	x	
Anger directed at the pupil		x
Anger directed at others		x
Social-image		x
Self-image		x

In line with previous research and theorizing, we expected that our community participants would regard a drop out as a wrong decision, and that the drop out is expected to hurt the family’s self and social-image. Importantly, based on the folk-view that a vocational education can be seen as a “second-chance” education, we anticipated the following results: firstly, we expected that a drop out from a vocational education would be seen to be making the family more upset, i.e., make them angrier at the former pupil, and angrier at others, than if the drop out had happened in a general education program. Secondly, anger directed at the former pupil would be explained by a concern for the family’s self-image. In contrast, anger directed at others would be explained by a concern for the family’s social-image.

## Materials and Methods

### Participants

Hundred and twenty nine community participants (62.2% women, 37.8% men; *mean age*: 36.1, *age range*: 17–74 years) agreed to partake in an anonymous, hard copy standardized questionnaire study focusing on social perceptions. They were approached individually in parks, cafes, and libraries in a medium-sized city in Norway. Participants were randomized into two conditions: “*Vocational education drop out*” (*N* = 64) and “*General education drop out*” (*N* = 65).

### Procedure and Measures

On the first page of the questionnaire participants read the information of the study as described above and agreed to partake in the study. On the same page, we asked the participant to fill in demographics of gender and age. On the next page, “*vocational education drop out*” participants were asked to imagine the following: “*A student at the (the name of a locally known vocational education high-school) decided to drop out from the education in the middle of the semester*.” Participants allocated to the “*General education drop out*” condition were asked to imagine the same thing, only now naming a locally known general education high-school. On the third page, participants were presented with standardized items measuring how this drop out could be appraised by the family of the student, and how they would respond to the drop out. When finished, participants were debriefed and thanked. All items were adopted from Gausel et al. (2012, 2016, 2018) and ranging from 1 (not at all) to 7 (very much).

#### Anger

*Anger directed at the pupil* (*α* = 0.96) was measured with: “The family would be angry at the pupil,” “The family would be cross at the pupil,” and “The family would be irritated at the pupil.” *Anger directed against those who know* (*α* = 0.93) was measured using three items: “The family would be angry at those who know what the pupil did,” “The family would be cross at those who know what the pupil did,” and “The family would be irritated at those who know what the pupil did.”

#### Appraisals of Social-Image and Self-Image

The appraisal of being condemned by others, and thus causing damage to the family’s *social image* (*α* = 0.87) was measured using three statements: “The family will think they can be isolated from others because of this,” “The family will think that their reputation can damaged because of what the pupil did,” and “The family will think that others might not have the same respect for them because of this.” The appraisal of damage to the family’s moral *self-image* (*α* = 0.89) was measured with three statements: “The family will think that what the pupil did represented a moral failure in the family,” “The family will think they are defective in one way or another,” and “The family will think this represents a “black mark” in their shared memory.”

#### Appraising the Drop Out as Wrong

We measured whether participants appraised the dropout as wrong (*α* = 0.85) using four items: “What the pupil did was wrong,” “What the pupil did was bad,” “What the pupil did was doubtful,” and “What the pupil did was not good.”

## Results

### Participants View of Dropping Out of School as Wrong or Not

A one way ANOVA using IBM SPSS 22 (see Table 2 for scale inter-correlations and descriptive statistics) made it clear that participants considered it wrong to drop out from college irrelevant of education, *F* (1,128) = 1.16, *p* = 0.28, *_partial_η* ^2^ = 0.01. Interestingly, they saw dropping out from the vocational education as slightly more wrong than from a general education (*M* = 4.28, *SD* = 1.67 and *M* = 3.97, *SD* = 1.54, respectively).

**Table 2:** Scale inter-correlations and descriptive statistics.

	Variable	1	2	3	4	5
1	Wrong decision	-				
2	Self-image (moral defect)	0.31	-			
3	Social-image (condemned by others)	0.17	0.69	-		
4	Anger directed at those who know	0.20	0.52	0.58	-	
5	Anger directed at the pupil	0.38	0.51	0.46	0.47	-
	Mean	4.13	2.53	2.40	1.77	3.20
	SD	1.60	1.42	1.35	1.14	1.69
	α	0.85	0.89	0.87	0.93	0.96

*N* = 129. Response scale ranged from *not at all* (1) to *very much* (7).

### A Concern for Self-Image and Social-Image

A *Multivariate ANOVA* demonstrated no significant overall effect on the appraisal of self-image and social-image, *F* (2,126) = 0.587, *p* = 0.56, *_partial_η* ^2^ = 0.01. A univariate analysis on each of the two variables showed that participants in the “*Vocational education drop out*” and the “*General education drop out*,” saw the drop out of school as equally damaging to the family’s self-image, *F* (1,127) = 1.16, *p* = 0.284, *_partial_η* ^2^ = 0.01 (*M* = 2.67, *SD* = 1.53 and *M* = 2.40, *SD* = 1.31, respectively), and the family’s social-image, *F* (1,127) = 0.73, *p* = 0.395, *_partial_η* ^2^ = 0.01, (*M* = 2.51, *SD* = 1.39 and *M* = 2.30, *SD* = 1.30, respectively) even though the means were a bit higher for participants in the vocational education drop out condition.

### Participants View on Anger Directed at the Pupil and Anger Directed at Others

A *Multivariate ANOVA* demonstrated an overall effect on our main dependent variables of anger, *F* (2,123) = 3.10, *p* = 0.049, *_partial_η* ^2^ = 0.05. As expected, there was a significant univariate effect on anger directed at others who would know about the drop out, *F* (1,124) = 4.51, *p* = 0.036, *_partial_η* ^2^ = 0.04. The pairwise comparison showed that participants in the “*Vocational education drop out*” condition considered it as more likely that the family would be angry at others who knew about the drop out (*M* = 1.97, *SD* = 1.27), than did participants in the “*General education drop out*” condition (*M* = 1.55, *SD* = 0.95). As expected, there was a significant univariate effect on anger directed at the pupil, *F* (1,124) = 4.53, *p* = 0.035, *_partial_η* ^2^ = 0.04. The pairwise comparison demonstrated that participants in the “*Vocational education drop out*” condition considered it likely that the family would be more angry with the pupil (*M* = 3.51, *SD* = 1.74), than did participants in the “*General education drop out*” condition (*M* = 2.88, *SD* = 1.58).

### Structural Equation Modeling: Explaining Direction of Anger

**Figure 1:**
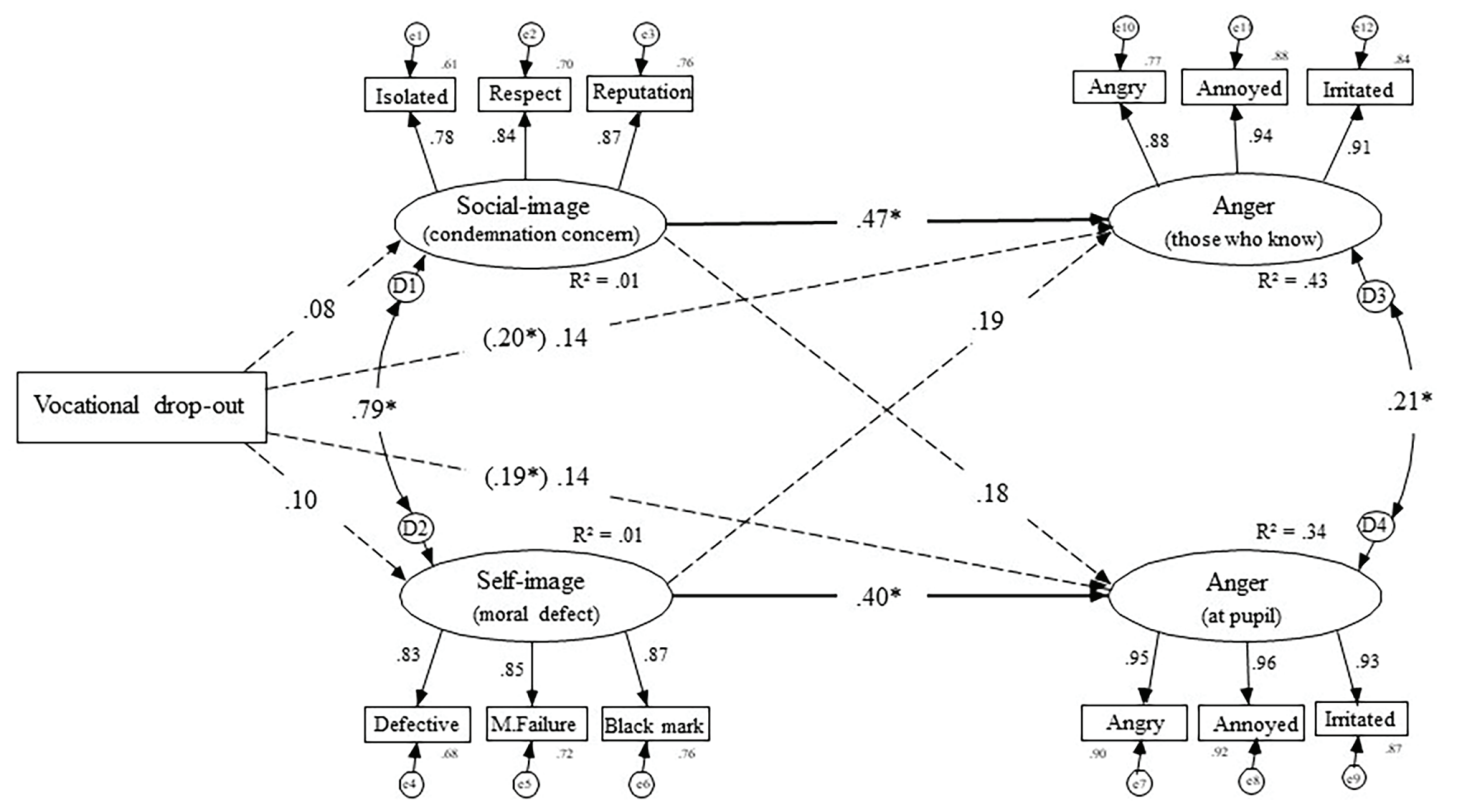
Structural path model of the manipulation on concerns for self-image and social-image and their link to the two angers. Solid lines illustrate significant relationships (^*^ *p* < 0.05).

In order to explain anger directed at the pupil and anger directed at others, we specified a latent model using *Structural Equation Modeling* with *AMOS 22* software. Mirroring the two conditions, we used effect coding (vocational education drop out = +1 and general education drop out = −1) in order to trace the main effects of the experimental conditions (represented with a manifest variable) on our two main dependent variables; anger directed at the self and anger directed at others. Since we expected a concern for the family’s self-image and concerns for the family’s social-image to explain the relationship with anger, we allowed them to mediate the relationship between the experimental conditions and the two anger variables (see Figure 1). This model fit the data very well, *χ* ^2^ (56) = 80.65, *p* = 0.017, *χ* ^2^/*df* = 1.44, *IFI* = 0.982, *CFI* = 0.982, *RMSEA* = 0.059.

As seen in the upper half of Figure 1, the original link between the experimental conditions and anger directed at the pupil (*β* = 0.19, *p* = 0.031) dropped to non-significant (*β* = 0.14, *p* = 0.077), indicating that the relationship was mediated by concern for the family’s self-image. In contrast, as we argued that the motivation behind anger directed at others was a concern for the family’s social-image, the lower half of Figure 1 illustrate that the original link between the experimental conditions and anger directed at others (*β* = 0.20, *p* = 0.032) dropped to non-significant (*β* = 0.14, *p* = 0.062). Hence, anger at others was mediated by concern for the family’s self-image.

## Discussion

Even though there can be good reasons for dropping out of an educational program, a drop out generally violates societal norms (e.g., Dorn, 1993; Gausel, 2014) such as the meritocracy norm of perseverance (Lerner, 1980) and taking advantage of the possibility to climb the social ladder *via* the educational system (Festinger, 1954; Hauser et al., 2000; Van Hoorn and Maseland, 2013). Probably therefore, our community participants considered dropping out to be moderately wrong regardless of the educational path, and by such, they lend support to Christle et al. (2007) view that a drop out represents a serious challenge, not only for the society but also for the school system, the community and the individual. Similarly, the decision to drop out was also viewed by the participants as a cause for concern in regard of both the family’s self-image and its social-image. This finding support Gausel and Leach (2011) argumentation that a failure to adhere to norms will likely threaten the self-image and the social-image of the individual (or group) associated with the failure.

In line with our hypotheses, we found that participants expected the family to be angrier at the former pupil for dropping out of vocational education than if dropping out of a general education. This is understandable, because expressing anger at the pupil might communicate that the family is disappointed over the decision to drop out of vocational education in an increasingly competitive labor market (Grootaers et al., 1999; Van Hoorn and Maseland, 2013). Moreover, since anger directed at the former pupil was explained by concern for moral self-image, the findings support the arguments of Gausel and Leach (2011) that a threat to self-image will likely motivate self-directed anger.

Also in line with our hypotheses, we found that participants in the vocational education condition expected the family to be angrier at others for the drop out than did those in the general education condition. As expected, the motivation to direct anger at others was explained by the concern for loss of respect in the eyes of others (i.e., the threat to the family’s social-image). This finding is in line with Gausel and Leach (2011) argument that the threat to the social-image is a motivator of anti-social responses and hostility. Moreover, this finding bears resemblance to Zahl-Olsen et al. (2019) findings where anger and violence in families seems to be fueled by rejecting criticism for failure. It also lends support to Gausel et al. (2018) findings that victims of failures reacted with hostile anger toward others due to the fears that their social-image would be damaged. By such, it appears that the community participants expected reactions similar to those reported in recent research and theorizing on anger and anti-social motivations.

### Possible Limitations

It should be underlined that our study focused on how people in general think a family would respond to a drop out. Naturally, it would be ideal to investigate how actual families of those who drop out would respond to our research questions. Even though this might be seen as a more “natural” approach, it is useful to remember that the vignette method has been found to produce results equal to other ecological methods (Robinson and Clore, 2001) only without the ethical dilemmas attached with real-world challenges. Moreover, as people are good at imagining how others and themselves would feel and do in various situations (e.g., Decety and Grèzes, 2006), the vignette design seems to be a useful tool on topics such as failures and how to cope with them.

That said, one should be aware of the practical and ethical difficulties to find and locate families with pupils that have dropped out of school. In relation to the practical difficulties of locating them, we can inform that we first tried to contact the two different schools mentioned in our scenario in order to gain information about the drop outs. However, we were not granted this information and were thus left in the dark in response to locating these families. That said, out of ethical concerns, families of those who drop out might already have been exposed for stigmatizing attitudes and thus have experienced many emotional and practical hardships. One can imagine that if we were to locate them, it might not be welcomed if we were to address them about something they might very well be angry about.

Another limitation rests within the participant pool. We did not check if they had background from a vocational or a general education, and thus, we cannot guarantee that this would not have influenced their perception of drop out from the one or the other educational programs. Moreover, we did not ask for, and therefore could not control for whether their level of education influenced the results in any way. That said, we aimed for a randomized pool of community participants (instead of the more “normal” student participant pool) that were more or less mature participants with a mean-age of 36 years. We do believe that these participants have enough life-experience to be more moderate in their beliefs about the world than younger ones. Hence, we rest assured that the results based on the feedback from our participants can be trusted.

## Practical Thoughts

Our findings indicate that professional helpers working with drop outs might meet families that, ironically, communicate anger instead of gratitude for the help they are given. If so, it could be helpful to know that this anger is likely explained by their fear of condemnation and feared damage to their social-image as a respectable family for the “failure” to prevent their son or daughter from dropping out of an educational program. Moreover, if the family is angry at the former pupil then the professional helper might see that their anger can be explained by the worry that there is a moral failure within the family since they could not prevent the drop out. In any way, we think helpers can use our model to better understand how families cope with the social and family-related challenges that a norm violating drop out might represent.

## Data Availability Statement

The datasets generated for this study are available on request to the corresponding author.

## Ethics Statement

The studies involving human participants were reviewed through the standardized checklist of the Norwegian Centre for Research Data and found not to be subject to notification. Written informed consent from the participants’ legal guardian/next of kin was not required to participate in this study in accordance with the national legislation and the institutional requirements.

## Author Contributions

NG did the design and analysis and contributed to the interpretation of the data, theoretical framework and write-up, and approved submission. DB contributed to the interpretation of the data, theoretical framework and write-up, and approved submission. All authors contributed to the article and approved the submitted version.

### Conflict of Interest

The authors declare that the research was conducted in the absence of any commercial or financial relationships that could be construed as a potential conflict of interest.
